# Factors Associated With the Usability and Adoption of Continuous Monitoring Devices With Deterioration Alerting Systems in Acute Hospital Non‐ICU Settings: A Mixed Methods Study

**DOI:** 10.1155/jonm/3056495

**Published:** 2026-03-24

**Authors:** Jo-Fan Pan, David Wong, Kuan Liao, Dawn Dowding

**Affiliations:** ^1^ Division of Nursing, Midwifery and Social Work, School of Health Sciences, Faculty of Biology, Medicine and Health, The University of Manchester, Manchester, UK, manchester.ac.uk; ^2^ Leeds Institute of Health Informatics, School of Medicine, Faculty of Medicine and Health, University of Leeds, Leeds, UK, leeds.ac.uk; ^3^ School of Nursing, Faculty of Health and Social Sciences, The Hong Kong Polytechnic University, Hung Hom, Kowloon, Hong Kong SAR, China, polyu.edu.hk

**Keywords:** early warning scores, hospital, hospital units, monitoring, nursing staff, physiologic, telemetry, wearable electronic devices

## Abstract

**Aim:**

To identify factors associated with usability and adoption of continuous monitoring with deterioration alerting systems (CM‐DAS) in non‐ICU wards from clinicians’ perspectives.

**Background:**

Patient deterioration is a safety concern on general wards; intermittent vital sign checks can miss early decline. CM‐DAS can help, but impact depends on usability and clinician adoption, which remain variably achieved.

**Methods:**

Convergent mixed methods using the unified theory of acceptance and use of technology (UTAUT) model to guide data collection: An online UTAUT‐based survey (*n* = 111 clinicians, 20 countries; April–August 2023) and semistructured interviews (*n* = 10) were conducted. Quantitative data were analysed with nonparametric tests and composite PLS‐SEM (3000 bootstraps); qualitative data underwent thematic analysis; findings were integrated narratively.

**Results:**

Perceived usefulness and ease of use were positively associated with the intention to adopt CM‐DAS. In the multivariable PLS‐SEM, only intention to use the system (*β* ˜ 0.29, *p* ˜ 0.01) and prior CM‐DAS experience (*β* ˜ 0.28, *p* ˜ 0.01) were associated with routine bedside use; other constructs did not retain independent associations, and variance explained was modest (*R* ^2^_use≈0.15). Interviews corroborated benefits (patient safety and workflow) and highlighted barriers—false alarms, reliability/connectivity issues, technical language/user interface and gaps in support and training. Peer practices and patient/family responses shaped the climate for adoption.

**Conclusions:**

This study suggests that ensuring reliable infrastructure (signal stability, hospital Wi‐Fi and integration with EHR) is foundational for safe and sustained CM‐DAS operation. Routine use was most closely associated with clinicians’ intention to use the system and accumulated experience. Factors such as how easy a system is to use and how individuals perceived its usefulness strengthened an individual’s intention to use the system.

**Implications for Nursing Management:**

Management should prioritise reliable infrastructure, implement tiered alarm governance to reduce nonactionable alerts, designate ward super‐users supported by vendor service‐level agreements and deliver brief, recurring, practice‐embedded training so that intention translates into sustained, safe bedside use.

**Reporting Method**
 The study adhered to the GRAMMS reporting guidelines.

## 1. Introduction

### 1.1. Background

In‐hospital patient deterioration remains a critical global challenge in non–intensive care unit (non‐ICU) settings, where delayed recognition of worsening conditions leads to preventable adverse events, including cardiac arrests, unplanned ICU transfers and mortality [1]. Studies from eight countries estimate that 5%–10% of patients experience severe adverse events (SAEs) like cardiac arrests or ICU admissions during their hospital stay [1, 2]. In the UK, the authors in [3] estimated that 23% of in‐hospital deaths resulted from failures to recognise or respond to patient deterioration. Currently, intermittent vital sign observations and early warning score (EWS) systems are widely used by nurses to detect clinical deterioration. Their reliability in detecting deterioration is hindered by limited intermittent vital sign measurement, data inaccuracies and a delayed response [4, 5].

Continuous monitoring devices with deterioration alerting systems (CM‐DAS)​ have emerged as a promising solution by providing real‐time vital sign tracking and automated alerts to assist nurses in initiating prompt clinical interventions [6, 7]. These systems continuously measure key physiological parameters, typically via wearable sensors or bedside monitors, and generate immediate alerts when abnormal thresholds or trends are detected [6, 7]. Several reviews indicate that CM‐DAS can effectively reduce ICU admissions and mortality compared with standard intermittent observation and EWS [6, 7]. However, the evidence remains mixed, as some investigations report no significant improvements in patient outcomes [8, 9]. Consequently, the overall effectiveness of CM‐DAS in non‐ICU settings remains inconclusive.

One reason for these mixed outcomes may be the potential usability barriers in existing CM‐DAS, as multiple reviews have highlighted insufficient focus on these factors [6, 7, 8, 9]. Usability is defined as the extent to which specified users achieve goals with effectiveness, efficiency and satisfaction in a specified context of use [10, 11], and for medical devices, it is governed by usability‐engineering principles [12]. According to Coiera’s information value chain, achieving better outcomes depends on success across multiple stages: user interaction, information acquisition, decision‐making and subsequent changes in care processes [13]. Usability is especially critical during the interaction and information acquisition phases, where systems must provide accurate, actionable and timely data in an intuitive manner. Poor usability, such as manifesting as unclear interfaces, excessive false alarms or complex workflows, can cause clinician disengagement, diminish trust and undermine the system’s influence on clinical decisions [13, 14]. Alarm burden specifically is a recognised safety risk in hospitals and requires governance [15, 16]. In contrast, systems with high usability promote consistent utilisation, support effective decision‐making and integrate seamlessly into clinical workflows, ultimately enhancing patient outcomes. This lack of focus on usability in CM‐DAS design and implementation is a critical gap, as clinician acceptance and sustained engagement are pivotal to realising the transformative potential of CM‐DAS in non‐ICU settings.

### 1.2. Theoretical Framework

In order to systematically identify and analyse the factors influencing usability and adoption, this study adopts the unified theory of acceptance and use of technology (UTAUT) model, which provides a comprehensive framework for understanding key elements of technology use [17]. UTAUT identifies four key factors that are theorised to influence behavioural intention (BI) to use a technology as well as actual use.

(a) Performance expectancy (PE); the ability of the technology to provide benefits and enhance use performance
(b) Effort expectancy (EE); expectations about the ease of use of the technology
(c) Social influence (SI); the influence of others on whether the user starts and continues to use the technology
(d) Facilitating conditions (FC); the level of organisational and technical infrastructure that supports the use of the technology

Age, gender, experience and voluntariness of use (whether or not use is a choice or enforced) can coinfluence these relations [18].

The UTAUT model was applied to CM‐DAS in non‐ICU wards as follows. PE is the expected clinical benefit of CM‐DAS, such as earlier detection of deterioration and safer ICU‐to‐ward transitions. EE is the perceived ease of use, including clear screens and language, minimal cabling/portable form factors and manageable alerts. SI reflects norms and expectations from senior staff and peers, as well as patient and family reactions to alarms. FC are the organisational and technical supports that enable operation, including reliable devices and connectivity, responsive maintenance, EHR​ integration and structured training.

**FIGURE 1:**
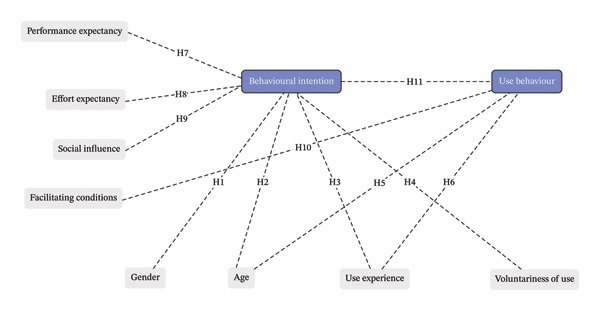
UTAUT conceptual model and hypotheses (H1–H11) tested in the study.

Consistent with UTAUT, we expected PE, EE and SI to be positively associated with BI; we expected FC to support actual use; we treated BI as the most proximal correlate of use. Because ward deployment often mixes voluntary and expected use and our sample was modest, age, gender, prior CM‐DAS experience and voluntariness were modelled as exogenous covariates (not moderators). In the structural model, age, gender, experience and voluntariness had direct paths to BI; age and experience also had direct paths to use behaviour. Figure 1 shows the adapted model; Section 1.3 lists the hypotheses (H1–H11); Section 2.4.1 details the operationalisation of the model.

### 1.3. Study Aim and Hypotheses

This study aimed to identify the factors influencing the usability and adoption of CM‐DAS in non‐ICU settings from clinicians’ perspectives.

## 2. Methodology

### 2.1. Study Design

A convergent mixed methods approach was used to address the study aims, collecting both quantitative and qualitative data simultaneously [19]. Quantitative data were collected from an online questionnaire that measured UTAUT factors influencing clinicians’ use of CM‐DAS and were designed to test the following hypotheses:

 H1. Gender is associated with BI (exploratory, two‐sided).
 H2. Age is associated with BI (exploratory, two‐sided).
 H3. Prior CM‐DAS use experience is positively associated with BI.
 H4. Perceived voluntariness of use is positively associated with BI.
 H5. Age is associated with use behaviour (exploratory, two‐sided).
 H6. Prior CM‐DAS use experience is positively associated with use behaviour.
 H7. PE is positively associated with BI.
 H8. EE is positively associated with BI.
 H9. SI is positively associated with BI.
 H10. FC are positively associated with use behaviour.
 H11. BI is positively associated with use behaviour.

Note: In the original UTAUT, age, gender, experience and voluntariness act mainly as moderators; in this study, they were included as direct predictors (covariates) of BI—and age and prior experience were also direct predictors of use behaviour; no moderation (interaction) effects were estimated—see Section 2.4.1 for operationalisation details.

Qualitative semistructured online interviews offered deeper insights into individual experiences and perceptions, capturing factors such as personal motivations and subjective attitudes. Quantitative and qualitative data were combined to produce a comprehensive understanding of clinicians’ experiences [19].

### 2.2. Study Procedure

#### 2.2.1. Recruitment and Sampling

Clinicians, including nurses, nurse assistants and physicians, with experience using CM‐DAS in adult non‐ICU wards within the past 3 years were considered for inclusion for this study. Physicians were deliberately included to expand the potential sample and to capture additional perspectives from those who may have interacted with CM‐DAS in their clinical practice, even if their involvement was less direct than that of nursing staff. From April to August 2023, a snowball sampling strategy was employed via social media platforms (Facebook, Twitter and LinkedIn) inviting participants to complete an online questionnaire. Upon completing the online questionnaire, participants could volunteer for a follow‐up one‐on‐one interview via Microsoft Teams.

#### 2.2.2. Quantitative Data Collection (Online Questionnaires)

Participants first accessed a self‐administered online questionnaire created via Qualtrics. The questionnaire was adapted from [17] to suit the study’s objective (Table S1 in supporting information). A description was added before the questions to clarify that the system being discussed refers to CM‐DAS in non‐ICU settings. Each construct from the UTAUT was assessed using four items, while BI was measured with three items. All items were rated on a 5‐point Likert scale (1 = *strongly disagree* and 5 = *strongly agree*) [20]. Context variables (age, gender, use experience and voluntariness of use) were also recorded [18]. Completing the questionnaire typically took 10–15 min.

#### 2.2.3. Qualitative Data Collection (Semistructured Interviews)

The first 10 eligible respondents from the survey who agreed to participate were selected based on previous sample size estimates for interviews. An interview protocol (Table S2 in supporting information), pilot‐tested with a doctoral‐level nurse, ensured clarity and coverage of key usability factors. The questions explored clinicians’ perceptions of CM‐DAS effectiveness, ease of use, social/organisational influences and required supports or resources. Each participant was interviewed once, with each interview lasting approximately 30–40 min; interviews were audio‐recorded with participant consent and subsequently transcribed anonymously. A £15 e‐voucher was provided as a token of appreciation. FC were probed through prompts on infrastructure reliability (e.g., signal and Wi‐Fi), device availability, maintenance responsiveness, training and electronic health record (EHR) integration.

Although BI and use behaviour were key outcomes in the quantitative UTAUT‐based model, the qualitative strand was designed to elicit explanatory context for the determinants of BI and routine use (PE, EE, SI and FC) and to capture concrete examples of how these determinants manifested in day‐to‐day practice. BI and use behaviour were therefore measured primarily via the survey, and the interviews were used to explain and contextualise the quantitative associations.

### 2.3. Ethical Considerations

Ethical approval was obtained from The University of Manchester Proportionate UREC (Ref: 2023‐16166‐28009). All data were stored on secure, university‐approved platforms, and transcripts were prepared by vetted University of Manchester contractors.

### 2.4. Data Analysis

#### 2.4.1. Quantitative Analysis

The quantitative analysis examined how UTAUT constructs relate to clinicians’ BI and how BI and contextual factors relate to actual use of the system. A composite‐based partial least squares structural equation model (PLS‐SEM) was selected because it (i) estimates all paths simultaneously in theory‐driven models with several correlated predictors, (ii) performs well with modest samples and non‐normal item distributions, and (iii) provides bootstrap confidence intervals for path estimates [21]. All paths are interpreted as associations; the cross‐sectional design does not support causal inference.

Consistent with the adapted UTAUT (Figure 1; Section 1.3), the model assessed whether clinicians reporting higher PE, EE, SI and perceived voluntariness also reported higher BI. It then assessed whether use behaviour was associated with BI, FC, age and prior CM‐DAS experience. In this adaptation, age, gender, prior experience and voluntariness were included as direct predictors (covariates) of BI, and age and prior experience were included as direct predictors of use behaviour; moderation (interaction) effects were not estimated because use was partly mandated, and the sample was modest.

Survey responses were exported from Qualtrics, incomplete or invalid cases were removed and Likert items were coded 1–5. Descriptive summaries were produced for participant characteristics and items. As a descriptive check of zero‐order relationships (i.e., before mutual adjustment in the model), Spearman correlations were calculated among age, prior experience, perceived voluntariness, UTAUT constructs, BI and use behaviour, and the Kruskal–Wallis test compared BI by gender [22]. These preliminary summaries contextualised the multivariable model; inference was based on the bootstrapped structural model. Estimation and reporting were conducted as follows: Reflective (Mode A) measurement was specified for PE, EE, SI, FC, BI and use behaviour. Models were bootstrapped with 3000 resamples. Results are reported as standardised path coefficients (β) with 95% bootstrap confidence intervals, *p* values, and *R* ^2^ for BI and use behaviour.

Internal consistency was evaluated using Cronbach’s *α* and composite reliability (CR ≥ 0.70); convergent validity using average variance extracted (AVE ≥ 0.50) and indicator loadings; and discriminant validity using the Fornell–Larcker and the HTMT ratio (< 0.85–0.90) [23, 24]. Inner‐model VIFs were examined to screen for collinearity; a conservative threshold (< 3.3) was applied with awareness that VIF < 5 is commonly acceptable. For the two‐item use behaviour scale, internal consistency is summarised by composite reliability and interitem correlation (*α* is not recommended for *k* = 2).

The analysis first confirms that the measurement of each construct is reliable and valid; it then interprets the structural paths. In plain terms, do higher perceived benefits, ease‐of‐use and social norms align with higher intention? And, after accounting for those beliefs and background factors, is routine bedside use most closely aligned with intention and prior hands‐on experience, with FC acting as enablers of operation?

#### 2.4.2. Qualitative Analysis

A thematic analysis approach was used to examine clinicians’ perceptions and experiences with CM‐DAS. Following Braun and Clarke’s established method [25], the first author (J.F.P.) led the coding process by reviewing the transcripts multiple times and applying initial codes in NVivo 12 [26]. These initial codes were organised into themes based on the UTAUT constructs. Codes that fell outside these constructs were grouped into new themes, ensuring comprehensive coverage of participants’ insights. Each theme was checked to ensure that all data within it fit together meaningfully, while also being clearly distinct from other themes [27]. Where overlaps or inconsistencies were identified, the themes were modified to improve clarity and coherence. Representative quotations were selected to illustrate the findings, providing a rich, contextual understanding of clinicians’ experiences and supporting subsequent integration with quantitative results. Finally, the coauthors (D.D. and D.W.) independently reviewed the coded data for accuracy and consistency. Any discrepancies were discussed and resolved through consensus, resulting in the refinement of codes and the creation of coherent themes.

Trustworthiness and rigour were addressed using established qualitative criteria (credibility, transferability, dependability and confirmability) [28, 29]. Credibility was strengthened through triangulation between the survey and interview strands and through iterative peer debriefing with coauthors during coding, with discrepancies discussed and resolved by consensus. Transferability was supported by reporting participants’ characteristics and study context. Dependability and confirmability were supported by maintaining an audit trail of analytic decisions and systematic coding procedures in NVivo; representative quotations are presented to demonstrate transparency between the data and the themes [30].

#### 2.4.3. Integration of Findings

At the interpretation and reporting level, narrative weaving was used for integration [31], and reporting followed the GRAMMS guidance for mixed‐methods studies [32]. Integration outcomes were assessed as confirmation, expansion or discordance; in the latter case, potential sources of bias were considered, data reexamined, and literature consulted. Specifically, qualitative themes were used to interpret the survey‐derived BI and use behaviour results (including nonsignificant paths) rather than to remeasure BI/use behaviour qualitatively.

## 3. Results

### 3.1. Quantitative Findings

A total of 249 participants started the survey, with 111 completing it (45.0%). Over half were aged 20–29 years (52%), and 72% were female. The majority had 2–5 years of CM‐DAS use experience (36%) and 2–5 years of overall clinical experience (42%). Most respondents were nurses (75%), reflecting their predominant role in device usage. Nearly 80% expressed willingness to use CM‐DAS. The majority held a bachelor’s degree (64%). Participants represented 20 countries, with 32% from Taiwan. Country proportions with Wilson 95% confidence intervals indicate that Taiwan contributed the largest share, with responses otherwise dispersed across 19 additional countries. Most respondents worked in general medicine (22%), emergency (16%) and general surgery (15%) (Table 1). In terms of device usage, 43% used CM‐DAS multiple times per week, and 24% reported daily use. The majority of respondents reported high willingness to use the devices voluntarily (Table 2). (Item‐level response distributions for the UTAUT scales [PE, EE, SI, FC and BI] are in Table S3 supporting information).

**TABLE 1:** Survey participant characteristics (*n* = 111).

Characteristics	Number of respondents (%) *n* = 111 (%)	
Gender (%)		
Male	28 (25)	
Female	80 (72)	
Other/prefer not to say	3 (3)	
Age (%)		
20–29 yrs	58 (52)	
30–39 yrs	43 (39)	
40–49 yrs	9 (8)	
5 0 and above yrs	1 (1)	
Education Background (%)		
High school diploma or equivalent	3 (3)	
Associate degree	2 (2)	
Bachelor’s degree	71 (64)	
Master’s degree	30 (27)	
Doctoral Degree	5 (4)	
Occupation		
Physician	16 (14)	
Nurse	83 (75)	
Health care assistant	4 (4)	
Other healthcare professional	8 (7)	
Respondents’ healthcare field experience (%)		
Less than 2 years	15 (14)	
2–5 years	47 (42)	
5–10 years	30 (27)	
More than 10 years	19 (17)	
Respondents’ medical speciality (%)		
Cardiology	7 (6)	
Emergency	18 (16)	
General internal medicine	24 (22)	
General surgery	17 (15)	
Neurology	8 (7)	
Obstetrics and gynaecology	9 (8)	
Oncology	5 (5)	
Others	23 (21)	
Country (%)		95% CI^a^ (%)
Taiwan	35 (32)	23.6–40.7
China (Mainland)	13 (12)	20.4–36.9
United States of America	11 (10)	7.0–19.0
Australia	9 (8)	5.6–16.9
United Kingdom of Great Britain and Northern Ireland	7 (6)	4.3–14.7
Singapore	5 (5)	3.1–12.4
Others	31 (28)	1.9–10.1

^a^95% CIs computed by the Wilson method (*z* = 1.96).

**TABLE 2:** Survey participant characteristics relevant to UTAUT constructs (*n* = 111).

Characteristics relevant to UTAUT constructs	Number of respondents (%)
	*n* = 111 (%)
Use experience^a^ (%)	
Less than 1 year	12 (11)
1–2 years	25 (22)
2–5 years	40 (36)
5–10 years	21 (19)
10 years above	13 (12)
Voluntariness of use^b^ (%)	
Very willing	47 (42)
Willing	42 (38)
Neutral	17 (15)
Unwilling	2 (2)
Very unwilling	3 (3)
Use behaviour (past 12 months)	
Daily	27 (24)
Frequently (multiple times a week)	48 (43)
Occasionally (once a week)	15 (14)
Rarely (once a month or less)	12 (11)
Never	9 (8)

^a^The experience of using CM‐DAS in non‐ICU settings.

^b^The degree to which the use of CM‐DAS in non‐ICU settings is perceived as voluntary or as an act of free will.

The UTAUT scales showed acceptable reliability and discriminant validity (Table 3). Convergent validity was borderline for SI (AVE = 0.46) and FC (AVE = 0.499), so results for these constructs should be interpreted cautiously. Collinearity in the structural model was low (all inner‐model VIFs 1.09–1.37) (full statistics are in supporting files Table S4–S6). BI was higher among clinicians who rated CM‐DAS as more useful (H7), easier to use (H8), and more socially supported (H9), and among those who perceived greater voluntariness (H4) (all *p* ≤ 0.008). BI and prior CM‐DAS experience were each positively associated with self‐reported use (H11 and H6). Intention did not differ by gender (H1 not supported). Only BI (H11, *β* = 0.288, 95% CI 0.055–0.560, *p* = 0.011) and prior CM‐DAS experience (H6, *β* = 0.276, 95% CI 0.071–0.485, *p* = 0.012) independently predicted routine use. Perceived usefulness, ease of use, SI, voluntariness, age and gender did not show independent associations with intention after mutual adjustment (H1–H2, H4 and H7–H9 not supported in the structural model), and FC and age did not predict use (H10 and H5 not supported). Explained variance was modest (*R* ^2^ ˜ 0.19 for intention; *R* ^2^ ˜ 0.15 for use). In a cohort that generally viewed CM‐DAS as useful and easy to use, actual routine use was most closely associated with BI and prior hands‐on experience; other beliefs aligned with intention in simple tests but did not add unique explanatory value once considered together.

**TABLE 3:** Hypothesis tests and structural paths (composite‐based PLS‐SEM).

H	Path	Bivariate (r/KW)	β (std)	95% CI low	95% CI high	Bootstrap p	Supported?
H1	Gender PE ⟶ ^a^behavioural intention	KW *p* = 0.438	0.071	−0.086	0.233	0.326	No
H2	Age PE ⟶ behavioural intention	*r* = −0.048, *p* = 0.619	−0.023	−0.184	0.166	0.901	No
H3	Use experience PE ⟶ behavioural intention	*r* = −0.026, *p* = 0.783	−0.051	−0.263	0.133	0.566	No
H4	Voluntariness of use PE ⟶ BI	*r* = 0.379, *p* = 0.000	0.174	−0.019	0.416	0.077	No
H7	PE PE ⟶ BI	*r* = 0.415, *p* = 0.000	0.170	−0.017	0.455	0.081	No
H8	EE PE ⟶ BI	*r* = 0.252, *p* = 0.008	0.058	−0.177	0.238	0.593	No
H9	SI PE ⟶ BI	*r* = 0.314, *p* = 0.001	0.230	−0.049	0.431	0.102	No
H5	Age PE ⟶ Use behaviour	*r* = 0.118, *p* = 0.219	0.041	−0.145	0.226	0.645	No
H6	Use experience PE ⟶ Use behaviour	*r* = 0.287, *p* = 0.002	0.276	0.071	0.485	0.012	Yes
H10	FC PE ⟶ Use behaviour	*r* = −0.006, *p* = 0.952	−0.085	−0.298	0.126	0.395	No
H11	BI PE ⟶ Use behaviour	*r* = 0.241, *p* = 0.011	0.288	0.055	0.560	0.011	Yes

*Note:* Model variance explained: *R* ^²^(BI) = 0.193; R²(use behaviour) = 0.148.

^a^The arrow ‘⟶’ means from predictor to outcome (e.g., PE ⟶ BI tests the path from performance expectancy to behavioural intention).

### 3.2. Qualitative Findings

**FIGURE 2:**
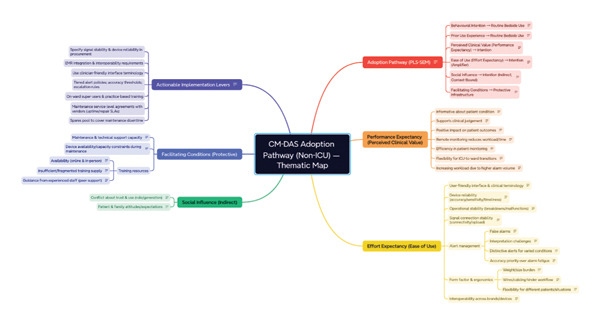
Thematic map linking qualitative themes to the quantitative adoption pathway. Perceived value strengthens intention; intention and prior use experience most proximally predict routine use. Ease of use acts as a conditional amplifier; social influence is indirect; facilitating conditions protect rather than propel use. Actionable levers (tiered alerts, clinical terminology in UI, uptime/maintenance SLAs, super‐users and EHR integration) are annotated.

A total of 10 online interviews were conducted; participants’ characteristics are presented in Table S7 in supporting files. Table 4 provides an overview of the themes with frequency of responses, and Figure 2 provides an overview of the thematic map of qualitative findings.

**TABLE 4:** Qualitative codebook with relative frequencies (*n* = 10 interviews).

Theme	Subtheme	Code	Description	Participants	*n* (%)
Effort expectancy	Alert management	Accuracy priority	Accuracy of alarms prioritised over alarm fatigue concerns.	P1, P3, P5, P6	4 (40.0)
		Distinctive alerts for varied conditions	Need for different alarms for different conditions to improve monitoring.	P5, P6	2 (20.0)
		False alarm	Alarms do not represent the patient’s condition.	P6, P7, P8, P9, P10	5 (50.0)
		Interpretation challenges of alarm	Alarms lack clear explanations; interpretation is challenging.	P1, P2 ,P3, P5	4 (40.0)
	Device reliability	Monitor reliability	Concerns about device accuracy/sensitivity; need for trustworthy, timely readings.	P1, P3, P4, P5, P6, P10	6 (60.0)
		Operational stability	Frequent breakdowns/malfunctions undermine stability.	P2, P3, P4, P6, P8, P9, P10	7 (70.0)
		Signal connection stability	Connectivity issues; vital signs fail to upload.	P1, P5, P10	3 (30.0)
	Ease of use design	Compatibility between different devices	Need for interoperability across brands/devices.	P2, P5, P7, P8, P10	5 (50.0)
		Flexible design for different patients	Devices lack adaptability to different patients/situations.	P1, P2, P4, P5, P6, P8, P9, P10	8 (80.0)
		Negative experience with device weight and size	Large size/heavy devices hinder use.	P2, P5, P7, P9, P10	5 (50.0)
		Negative experience with device wires	Wires tangle and slow work.	P1, P3, P4, P7	4 (40.0)
		User‐friendly interface design	Need for an intuitive UI, clinician‐friendly terminology.	P1, P2, P3, P4, P5, P6, P7, P8, P9, P10	10 (100.0)

Facilitating conditions	Maintenance	Limited device capacity	Insufficient device availability during maintenance/unavailability.	P3	1 (10.0)
		Limited technical support team	Limited maintenance/technical support access.	P1, P3, P4, P6, P7, P10	6 (60.0)
	Training	Availability of training resources	Online and in‐person training resources are available.	P2, P4, P5, P6, P7, P10	6 (60.0)
		Complained about insufficient training resources	Training resources are limited; need for structured programs.	P1, P3, P5, P9, P10	5 (50.0)
		Guidance from experienced staff	Rely on peer guidance when issues arise.	P3, P6, P8, P9, P10	5 (50.0)

Performance expectancy	Assisting in patient monitoring	Flexibility in ICU‐to‐ward transitions	Supports transitions between ICU and general wards.	P2, P7	2 (20.0)
		Informative in‐patient condition	Provides effective information on patient condition.	P1, P3, P4, P6, P7, P8, P9, P10	8 (80.0)
		Positive impact on patient outcomes	Earlier detection and positive impact on outcomes.	P2, P4, P6, P7, P8, P10	6 (60.0)
		Support for clinical judgement	Assists decision‐making and clinical judgement.	P1, P4, P5, P6, P7, P8, P10	7 (70.0)
	Personal belief and trust	Personal belief and trust	Voluntariness/openness to technology influence use.	P2, P5, P7, P8	4 (40.0)
	Workload impact	Efficiency in patient monitoring	Improves efficiency; saves time.	P1, P2, P3, P4, P5, P6, P7, P8, P9, P10	10 (100.0)
		Increasing workload due to more alarms	More alarms increase response workload.	P1, P7	2 (20.0)
		Workload reduction via remote monitoring	Remote monitoring reduces workload/time.	P1, P2, P3, P4, P6, P7	6 (60.0)

Social influence	—	Conflict about trust and use	Generational/role‐based conflict over trust and adoption.	P2, P10	2 (20.0)
		Patient and family attitude	Patient/family anxiety/expectations influence use.	P3, P7, P8, P9, P10	5 (50.0)

*Note:* ‘Participants (P#)’ are derived from the referees in the thesis; *n* (%) reflects the number (percentage) of interviewees mentioning each code. Denominator *n* = 10 interviews (P1–P10).

#### 3.2.1. Theme 1: PE—Balancing Clinical Benefits and Workflow Realities

Clinicians perceived continuous monitoring devices as tools to enhance patient safety through real‐time surveillance while navigating tensions between efficiency gains and workflow disruptions. Participants emphasised the devices’ ability to fill critical gaps in non‐ICU care, with one nurse explaining,

‘We know the patient’s situation, and we can record the patient’s vitals, so at least we can see any deterioration, and also the heart rate all the time. Also, we can monitor the patient’s blood pressure very frequently, which are the benefits.’ (P10)

This real‐time data empowered clinicians to manage higher‐risk patients in general wards, particularly those transitioning from intensive care. As one participant noted,

‘They don’t really need to stay in ICU, but they are not really stable that we can keep them in a normal room.’ (P2)

While many highlighted workload reductions through automated documentation *(‘It’s really helped us to save a lot of time especially that you don’t need to worry about whether you will write the first patient’s data to the third patient’* [P2]), some described unintended consequences. Alarm fatigue emerged as a paradoxical challenge: devices designed to streamline care occasionally intensified workloads.

“These machines add to our burden… alarms ring constantly based on patient conditions” (P1),

though others anticipated long‐term efficiency gains. A participant speculated,

“In the long term, staff could have more face‐to‐face time instead of running for routine obs” (P7),

suggesting initial disruptions might yield future benefits. Trust in the technology’s clinical value strongly influenced adoption. Clinicians who viewed the devices as reliable partners in care—rather than passive tools—reported greater willingness to integrate them into practice. One nurse tied this to personal philosophy:

“I believe new techniques often improve care—I’m willing to learn” (P2).

For these adopters, positive outcomes reinforced trust, creating a self‐sustaining cycle of acceptance.

#### 3.2.2. Theme 2: EE—Navigating Usability Challenges in Design, Alerts and Reliability

Clinicians underscored that the perceived ease of using CM‐DAS hinges on balancing intuitive design, reliable performance and actionable alerts. While acknowledging their potential, participants highlighted systemic barriers complicating adoption in non‐ICU settings.

A central tension emerged around alert systems. Though recognised as vital for patient safety, frequent false alarms strained workflows. One nurse described,

*“When patients walk in* the *hallway, alarms trigger… on a busy floor, it makes our work heavier”* (P6).

Alarm fatigue risked desensitising staff, yet clinicians conceded their necessity:

*“Even annoying,* these *alarms are important signs to observe patient”s”* (P1).

To mitigate this, participants advocated for tiered alerts distinguishing urgency levels.

*“Non-emergent* alarms *shouldn’t sound like emergencies—different colors could help us prioritize”* (P5).

Complexity compounded the issue; some alerts required specialised knowledge, creating care delays.

“If the output is too complex for nurses to read, we’re stuck—doctors aren’t always nearby” (P3).

Device reliability further tested clinicians’ patience. Wearables, praised for portability, faced connectivity hurdles. *‘Wireless devices would be useful if signals improved’* (P1), remarked a nurse, contrasting them with stationary monitors’ stability. Yet even traditional systems faltered:

*“Sometimes the* monitor *blacks out… you know something’s wrong”* (P3).

Bedside devices, though reliable, posed physical challenges—bulky designs disrupted mobility.

“Dragging heavy monitors to scans is problematic… wearables are better, but long‐term use still feels burdensome” (P9).

Design intuitiveness profoundly shaped adoption. Clinicians criticised interfaces cluttered with engineering jargon.

*“Labels should* use *medical terms—not confusing technical language”* (P1).

Iterative codesign improved usability, as one individual noted

*“We revised interfaces based on feedback until they were easy to navigate”* (P7).

Physical design also mattered: tangled wires (*‘They make work less efficient’* [P1]) and poor patient adaptability (*‘Dementia patients might harm themselves with devices’* [P10]) highlighted mismatches between technology and care realities.

System compatibility emerged as a hidden burden. Fragmented technology ecosystems forced clinicians to relearn workflows.

“Switching brands *means* buying all new sensors—it’s inefficient” (P2).

Familiarity eased this strain; one participant likened ideal devices to *‘something intuitive, like an iPhone’* (P7), underscoring the need for seamless integration into existing routines.

#### 3.2.3. Theme 3: SI—Navigating Generational Divides and Patient Perceptions

Clinicians described how social dynamics—spanning intergenerational workplace attitudes, patient responses, and family expectations—shaped their engagement with continuous monitoring devices. Resistance to technological change often stemmed from generational divides, with younger staff more readily embracing innovation than senior colleagues.

“Every time the hospital introduces a new system, no one likes it—you have to relearn everything” (P2)

noted one participant, highlighting inertia rooted in workflow disruption. This tension extended to leadership influence; senior clinicians’ minimalist use of devices *(‘They just use the machine to take notes…we don’t think about improving’ [P10])* inadvertently discouraged deeper integration among junior staff.

Patient and family reactions further complicated adoption. Devices designed to reassure sometimes heightened anxiety, particularly among vulnerable populations.

“Dementia patients get agitated—the alarms are noisy, and they might harm themselves” (P10)

explained a nurse, while families misinterpreted routine alerts as emergencies.

“Families complain about the constant beeping…it’s stressful for them” (P8).

These experiences underscored a paradox: Tools intended to foster trust could erode it when usability clashed with human‐centred care.

Yet participants also reflected on bridging these divides. One clinician, self‐identifying as ‘willing to try high‐tech despite being in my 40s’ (P2), framed openness as a mindset rather than an age‐dependent trait. Their stance hinted at pathways to reconcile tradition with innovation—balancing the scepticism of experienced staff with the adaptability of newer generations, while addressing patient and family concerns through clearer communication.

#### 3.2.4. Theme 4: FC—Bridging the Gap Between Support Structures and Clinical Realities

Clinicians stressed that successful integration of continuous monitoring devices in non‐ICU settings depends on robust institutional support yet highlighted systemic gaps in maintenance and training that undermine their potential.

Maintenance challenges emerged as a critical barrier. While devices required consistent upkeep, participants described fragmented technical support.

“There’s no dedicated team readily available—we often reboot devices ourselves” (P10)

shared one nurse, illustrating makeshift solutions like power‐cycling equipment to avoid delays. Hospital maintenance teams, stretched thin across diverse technologies, struggled to provide timely expertise.

“Calling IT takes time… meanwhile, patient monitoring stops” (P10).

This reactive approach left clinicians balancing patient care with improvised troubleshooting, eroding trust in system reliability. Training programs, though available, faced similar tensions. While some hospitals offered on‐demand workshops *(‘Teams come to train staff upon request’ [P4]*), participants emphasised superficial or inconsistent instruction.

“Training feels rushed—it’s delivered ‘in the moment,’ not comprehensively” (P5).

To bridge this gap, some institutions incentivised engagement, as one team noted:

“Offering professional development points boosted nursing attendance” (P7).

Yet these efforts often clashed with workflow demands, pushing clinicians toward informal learning.

“When devices fail, seniors just tell us to swap them out… no real troubleshooting” (P10)

underscoring reliance on peer guidance over structured education. The interplay of these issues revealed a paradox: Tools designed to enhance care risked becoming burdens without aligned support. Clinicians navigated a patchwork of DIY fixes and fragmented learning, echoing broader systemic divides between technological ambition and on‐the‐ground resourcing. As one participant reflected, *‘You need more than devices—you need a culture that maintains and trains’ (P10)*, advocating for solutions that pair innovation with institutional accountability.

### 3.3. Integration of Findings

In the adjusted quantitative model, only BI and prior CM‐DAS experience were independently associated with routine bedside use, whereas perceived usefulness, ease of use, SI, FC, age and gender were not. The qualitative accounts help explain this pattern. Nurses described how repeated, hands‐on exposure builds familiarity and confidence, leading to trust in trend data, practical workarounds and shorter workflows, so that initial willingness to use the system develops into automatic, everyday practice. Perceived clinical benefits and ease of use were said to make clinicians willing to adopt the technology, but on their own did not sustain day‐to‐day use without that accumulated experience. SIs were experienced as the surrounding climate, for example, senior norms, peer example and patient/family reactions, which shaped expectations rather than directly driving use. FC, such as having a reliable signal, responsive maintenance of the devices, integration with EHR systems and on‐ward training, were viewed as prerequisites that enable the system to run safely. When these conditions were not present, use stalled despite good intentions. Taken together, the mixed‐methods evidence suggests a pathway in which perceived benefits and ease of use inform intention to use CM‐DAS, and this intention coupled with accumulating experience, in a supportive environment, is what translates into routine use. Table 5 summarises how the qualitative themes illuminate the quantitative pattern and the implementation levers this implies for nurse managers.

**TABLE 5:** Integrated findings and implementation levers (joint display).

UTAUT construct	Everyday practice signals	Role in the adoption pathway	Integration judgement	Actionable levers (non‐ICU)
Performance expectancy	Real‐time surveillance; earlier detection; smoother ICU⟶ward transitions; perceived clinical value	Shared backdrop that strengthens intention rather than a stand‐alone driver	Confirm	Specify trend accuracy; visualise deterioration trajectories; include outcome‐relevant use cases in onboarding
Effort expectancy	Wiring/portability; alert salience; terminology; interface layout	Conditional amplifier of intention (low friction reinforces; high friction undermines)	Expand	Tiered alerts; clinician‐friendly UI language; minimise cables/footprint; co‐design screens with ward nurses
Social influence	Senior‐staff norms; peer practice; patient/family reactions to alarms	Indirect, context‐bound influence via culture and perceived risk	Expand	Visible senior use; super‐user/peer mentoring; patient/family briefings on alarm meaning/escalation
Facilitating conditions	Maintenance responsiveness; integration with existing systems; training cadence	Supportive substrate that protects the pathway rather than driving it	Discord	Uptime SLAs; integration latency targets; on‐ward super‐user coverage; scheduled refreshers; rapid swap/loaners
Behavioural intention	Willingness to use aligned with patient‐safety goals	Proximal conduit converting beliefs into action	Confirm	Align local KPIs to safety use cases; audit and feedback huddles on appropriate use
Use experience	Familiarity; troubleshooting competence; reduced cognitive load; routine incorporation	Proximal driver of sustained, routine use	Confirm	Practice‐embedded training; just‐in‐time job aids; staged alarm policies; simulation of common failure modes

## 4. Discussion

The aim of this study was to investigate the factors influencing nurses’ adoption of a CM‐DAS on general (non‐ICU) wards. It highlighted that nurses’ intention to use the system was shaped by perceived usefulness (PE), ease of use (EE), SI and FC (such as reliable signal/Wi‐Fi, responsive maintenance, EHR integration and practice‐embedded training). These factors were a strong determinant of actual usage, together with prior experience with similar technology [18, 17, 33]. Nurses interviewed in our study recognised the potential of continuous vital sign monitoring to enhance patient safety (for example, by enabling earlier detection of deterioration), in line with literature showing real‐time surveillance can support earlier recognition of clinical decline on general wards [4, 7, 9]. However, they also raised concerns about practical barriers, most notably alarm fatigue and workflow integration, echoing known challenges in implementing new monitoring technologies [15, 34, 35]. The following discussion weaves together the quantitative and qualitative findings to explore these points in detail and their management implications.

Perceived usefulness (PE) emerged as a pivotal driver of intention to use CM‐DAS. Quantitatively, PE correlated with stronger intention to use CM‐DAS, though the association did not retain an independent effect in the multivariable model. This aligns with the UTAUT framework and meta‐analytic evidence that perceived usefulness is a primary motivator of healthcare IT acceptance [18, 33, 36]. For example, staff have been enthusiastic about continuous ward monitoring because they feel it allows earlier identification of patient deterioration and provides reassurance to patients and clinicians [37, 38]. Our qualitative data mirrored this sentiment, with many nurses valuing CM‐DAS for the ‘peace of mind’ it offered, noting cases where the system’s alerts could act as an early warning and potentially prevent adverse events. By sharing such success stories and peer‐reviewed evidence of clinical benefit with staff, managers can further strengthen the perceived usefulness of CM‐DAS for practice. Indeed, clearly demonstrating relative advantage, that is, how the new system improves upon usual intermittent monitoring, is critical for buy‐in [37, 7].

Perceived ease of use (EE) was another significant factor shaping nurses’ intentions to use the system from the survey results. If CM‐DAS was seen as user‐friendly and fitted smoothly into workflows, nurses reported higher intent to use it. If it was cumbersome or time‐consuming, hesitancy grew. This finding resonates with research showing that complexity and poor usability are consistent barriers to digital tool uptake in hospitals [14, 39]. A previous study [35] found that nurses identified system complexity and poor design (for example, tedious interfaces or multiple steps to get data) as strong negative influences on implementing continuous monitoring on wards. Similarly, extended‐UTAUT studies in healthcare show EE significantly influences professionals’ acceptance of telehealth solutions [36]. In our interviews, nurses highlighted practical difficulties such as managing sensor attachments, frequent false alarms and the learning curve of the software, all of which could increase the effort required to use CM‐DAS. These insights underscore that even a potentially beneficial system will face adoption hurdles if it is not designed and introduced with usability in mind. Thus, to promote adoption, hospital management should invest in training and support that boost users’ confidence and competence with the system and, wherever possible, simplify or refine the technology interface. Our results suggest that reducing perceived effort, through intuitive design, adequate training and integration into routine care, can significantly increase nurses’ willingness to consistently use the system.

SI and organisational context also affected nurses’ BI with staff who felt that their peers and supervisors supported or expected CM‐DAS use reporting stronger intentions to use it. This aligns with evidence that a positive unit culture and visible leadership support can facilitate the adoption of new nursing technologies [38, 40]. Indeed, strong leadership engagement is often cited as critical to foster a supportive climate for change, ensuring that nurses feel their use of the new system is valued and encouraged at all levels [38, 41]. In our qualitative data, the presence of ‘clinical champions’, respected nurses or doctors who advocated for the system and good communication within teams (such as sharing tips and early wins), was noted as a facilitator of CM‐DAS uptake. Conversely, if colleagues were sceptical or the system was not integrated into daily routines, individual nurses were more inclined to revert to familiar practices. Notably, SI in technology adoption can be intertwined with whether use is voluntary or mandated. As our measure captured perceived voluntariness (not organisational policy), we cannot infer whether use was voluntary or required. Still, leveraging social dynamics is important as creating a sense of collective endorsement (for example, via team feedback sessions or endorsements from senior nurses) can reinforce individual intentions to use the technology [41, 33].

FC, the practical resources and organisational supports available to users, were frequently raised as a concern during interviews, underscoring their importance for actual technology use. In our structural model, we included age, gender and voluntariness as covariates rather than UTAUT moderators, and none showed a significant direct effect on usage once core constructs and supports were accounted for. This suggests that with the right FC, nurses across different demographic groups can equally adopt the system. What mattered more were things like infrastructure reliability, training and ongoing support. For instance, nurses in the interviews pointed out that poor Wi‐Fi connectivity or insufficient availability of devices on the ward could severely impede CM‐DAS use. This aligns with [35] and related studies, where unreliable network connections and technical glitches were identified as major barriers to implementing wireless monitoring systems [35, 38, 40]. A recurring theme in our qualitative findings was that when technical or logistical issues arose (such as dropped signals or delayed alerts), it frustrated users and eroded trust in the system’s dependability. On the other hand, participants noted that having hands‐on training sessions and responsive technical support made them more confident to use CM‐DAS day‐to‐day. Our findings suggest that organisations should guarantee adequate IT infrastructure, staffing and ongoing technical support to back new digital tools [14, 39].

Our results also reinforce that BI is a strong predictor of actual use of new technology, as posited by UTAUT and related models. Nurses with higher self‐reported intention to use CM‐DAS were indeed more likely to report using the system in their daily practice ([17, 42, 36]; Table 3). Additionally, prior experience with similar monitoring technology significantly predicted use, with nurses who had previous exposure to continuous monitoring (for example, through a pilot, training simulator, or use of simpler telemetry) tending to use CM‐DAS more frequently, consistent with implementation studies where familiarity and self‐efficacy build over time [35]. Drawing on prior implementation work in ward‐based continuous monitoring, which highlights peer mentoring and practice‐embedded support during rollout, managers may designate experienced users as on‐ward ‘super‐users’ or champions to provide just‐in‐time assistance [38, 35, 40]. Our quantitative analysis showed that prior CM‐DAS experience was positively associated with routine use, suggesting experience is a plausible lever for uptake. Finally, we did not find meaningful differences by age or gender in the usage of CM‐DAS when support and training were in place, a pattern also reported in some healthcare UTAUT applications [36, 42]. Rather than demographic traits, our qualitative data point to modifiable supports such as training quality, peer guidance and perceived relevance as salient for uptake. This is an encouraging insight, suggesting that broad‐based uptake is achievable if the implementation strategy addresses users’ needs and concerns effectively.

The extent to which these findings translate to other hospitals and countries will depend on local conditions, particularly alarm governance, infrastructure/EHR integration and implementation approaches. In line with our qualitative findings on nonactionable alarms, sites that tailor alerts to the local environment—by tuning thresholds/delays, routing through existing tools and using watcher models—typically reduce nonactionable alarms and mitigate fatigue [15, 16, 34], whereas weak governance can amplify false alerts and discourage use. Trust and day‐to‐day usability also hinge on stable Wi‐Fi, adequate device/battery stock and smooth EHR connectivity with gaps undermining performance and confidence in the system [35, 38, 40]. Adoption is strengthened when procurement and training are participatory and practice‐embedded, involving codesign, on‐ward super‐users and availability of short refresher training. It is weakened by top–down mandates or workload pressure [38, 43, 44].

### 4.1. Limitations

Whilst the participants in the survey spanned 20 countries, there was overrepresentation of younger nurses and respondents from Taiwan; this constrains generalisability to other clinicians and settings. In addition, the survey could only be completed in English, meaning it excluded individuals who could not speak or understand that language. Recruiting individuals using social media means that the sample was self‐selected and included individuals who are already familiar with technology use. All quantitative data were self‐reported and cross‐sectional, so recall/social‐desirability bias is possible, and causal inference is not warranted; use behaviour was captured by brief self‐report rather than device logs. Measurement checks were acceptable overall, but convergent validity was borderline for SI and FC, and the two‐item use behaviour scale relied on composite reliability and interitem correlation, so findings for these constructs should be interpreted cautiously. Responses likely varied by context and device (brand, sensor form factor and EHR integration), and site/country determinants were not modelled, leaving potential residual confounding. The structural model used a modest sample (*n* = 111) and, for parsimony, treated UTAUT moderators as covariates, which may understate moderation effects. The qualitative component was small (*n* = 10), volunteer‐based and English‐language only, so less‐engaged users may be underrepresented. In addition, the interview protocol prioritised antecedent constructs, so BI and routine‐use experiences were not probed systematically; future qualitative work should include explicit questions on intention and actual use. Finally, outcomes and alarm/workload metrics were not measured; conclusions therefore address adoption and usability rather than effectiveness. Future studies should pair surveys with device telemetry and outcomes, model contextual factors, use multilingual instruments and test moderator effects in larger, device‐specific cohorts.

## 5. Implications for Nursing Management

For nurse managers and clinical leaders, the findings suggest that sustained use of continuous monitoring with deterioration alerts (CM‐DAS) is shaped less by individual characteristics and more by how implementation is supported in practice. Adoption is strengthened when staff can see a clear clinical benefit, experience a low burden of use and receive consistent encouragement from peers and senior staff, alongside reliable FC. Managers should therefore prioritise implementation conditions that reduce friction in day‐to‐day workflows. This includes ensuring robust connectivity and device availability, timely technical support and maintenance and clear escalation pathways for responding to alerts. Integrating CM‐DAS into routine documentation and handover processes (including electronic record integration where feasible) can help minimise duplicate work and improve staff confidence in the system’s outputs. To mitigate alarm fatigue and support safe use, ward leadership should implement agreed alert governance (e.g., threshold review, tiered prioritisation and regular audit of nonactionable alerts) and provide structured, ongoing training focused on interpretation, troubleshooting and clinical decision‐making. Establishing super‐users or peer champions, creating opportunities for sharing practical tips and success stories and monitoring implementation indicators (e.g., alert response time, user‐reported usability and sustained routine use) can support iterative improvement and long‐term normalisation of CM‐DAS in non‐ICU settings.

## 6. Conclusion

Clinicians’ routine bedside use of CM‐DAS on non‐ICU wards is most directly shaped by their intention to use the system and by prior hands‐on experience with similar tools. Perceived clinical value (e.g., real‐time trends and earlier detection) and ease of use (simple wiring/portability, clear alerts and intuitive language) strengthen that intention, while senior norms, peer practice and FC (reliable connectivity, EHR integration and accessible support) create the operational runway for safe use. Taken together, adoption depends less on persuasion alone and more on making the system dependable and straightforward at the point of care. In practice, organisations should ensure that purchasing decisions explicitly consider the reliability of CM‐DAS signal capture and how well the system fits with existing digital infrastructure, including seamless EHR integration. Alarm burden should be reduced by implementing tiered alarm‐governance policies that minimise nonactionable alerts. Local capability should be built by designating on‐ward super‐users who are backed by clear vendor service‐level agreements to guarantee timely support. Training should be embedded in routine workflow, delivered as brief refreshers with rapid troubleshooting so that confidence and competence translate into sustained bedside use. Generalisability is limited by English‐language, social‐media recruitment and heterogeneous devices and contexts. Future studies should incorporate objective usage telemetry and alarm analytics, deploy multilingual instruments and test adapted UTAUT models in larger, device‐specific cohorts.

## Author Contributions

Conceptualisation and methodology: Jo‐Fan Pan, Dawn Dowding and David Wong. Formal analysis, investigation, data curation, visualisation and writing–original draft: Jo‐Fan Pan. Writing–review and editing: Jo‐Fan Pan, Dawn Dowding, David Wong and Kuan Liao. Supervision: Dawn Dowding and David Wong.

## Funding

This research received no funding.

## Disclosure

No patients or members of the public were involved.

## Conflicts of Interest

The authors declare no conflicts of interest.

## Supporting Information

Supporting Information. Supporting Information File 1: Supporting Tables S1–S7—S1: Online questionnaire items and response options; S2: semistructured interview guide; S3: iem‐level response distributions for UTAUT constructs; S4: measurement model diagnostics; S5: HTMT matrix; S6: inner‐model collinearity (VIF); S7: interview participant characteristics and CM‐DAS usage patterns.

## Supporting information


**Supporting Information** Additional supporting information can be found online in the Supporting Information section.

## Data Availability

Deidentified survey data dictionary, codebook (Table S1) and analysis scripts used to generate summary tables are available from the corresponding author upon reasonable request. Qualitative transcripts are not shared publicly to protect participant confidentiality.
