# Supplementary material for: Factors Associated With the Usability and Adoption of Continuous Monitoring Devices With Deterioration Alerting Systems in Acute Hospital Non‐ICU Settings: A Mixed Methods Study
Source: J Nurs Manag. 2026 Mar 24;2026:3056495. doi: 10.1155/jonm/3056495 (PMC13140944; doi:10.1155/jonm/3056495)
Supplement: Supplementary file 1 — Supporting Information Additional supporting information can be found online in the Supporting Information section. [file JONM-2026-3056495-s001.docx]

**Supplementary Materials**

**Table S1 Online questionnaire (items and response options)**

| Section 1 Cover page | The cover page introduces the study, outlining its purpose, ensuring participant anonymity, and providing contact details for the researcher, supervisory team, and university for any inquiries. It concludes with a consent statement. |
| --- | --- |
| Section 2 Demographic background | Participants self-reported their demographics (gender, age, education, marital status, race/ethnicity, work experience) and technology usage, including frequency and willingness to adopt new technologies in various areas. |
| Section 3 Usability Questions | Venkatesh et al. (2003)’s UTAUT questionnaire with Likert 5-score scale. And A description points out the system refers to the following question is continuous monitoring devices with deterioration alerting in non-ICU settings. |
| Section 4 Interview Participation | To find out the potential participates to join our in-depth interview. |

**Table S2 Semi-structured interview guide**

| Part 1 Introduction of the study | |
| --- | --- |
| Introduction of the study to participant | Start with the title of the study, the purpose of the study, an explanation of anonymity, and contact information for the researcher, supervisory team, and university in case the participant has any questions. This is followed by an oral consent statement. |
| Part 2 General Questions | |
| Opening questions | - How would you describe your experience using a continuous monitoring system with deterioration alerting? - Can you tell me about your feelings towards the system? |
| Part 3 Questions | **Question description** |
| Performance expectancy | - How effective do you think the system is at detecting patients who are deteriorating? - How does the system affect your work as a healthcare professional? Does it make things easier or harder for you? |
| Effort expectancy | - How user-friendly do you find the design and layout of the system? - Have you encountered any technical issues while using the system? - Do you feel comfortable using the system, or have you encountered any challenges or difficulties? |
| Social influence | - Do you feel there is anyone impact your use of the continuous monitoring and deterioration alerting in a non-ICU setting? - Do you feel patient, or colleague impact your use of the continuous monitoring and deterioration alerting in a non-ICU setting? |
| Facilitating conditions | - How does the system fit into your work environment and daily routine? Do you feel it integrates well or are there challenges? - How well is the system supported and maintained by the organization? Do you feel you have the resources and support you need to effectively use the system? |
| Other Factors | - Are there any other factors that you believe impact your use of the continuous monitoring and deterioration alerting in a non-ICU setting? |
| Part 4 Ending | |
| Closing | - Final thoughts on the system - Thank the respondent for their time. - Provide contact information if they need to reach out to the organization regarding the study. |

**Table S3 Descriptive Data Analysis of UTAUT Constructs**

| Construct | Individual item | Measure | Number of Respondents, N=111, (%) | | | | |
| --- | --- | --- | --- | --- | --- | --- | --- |
|  |  |  | Strongly Agree | Agree | Neutral | Disagree | Strongly  Disagree |
| PE | PE1 | I find the continuous monitoring and deterioration alerting system useful in my job. | 47 (43) | 57 (51) | 6 (5) | 1 (1) | 0 (0) |
|  | PE2 | The use of the continuous monitoring and deterioration alerting system enables me to accomplish tasks more quickly. | 39 (35) | 51 (46) | 16 (14) | 3 (3) | 2 (2) |
|  | PE3 | The use of the continuous monitoring and deterioration alerting system helps me to better manage my patients' conditions. | 57 (51) | 48 (43) | 6 (5) | 0 (0) | 0 (0) |
|  | PE4 | Using the continuous monitoring and deterioration alerting system could enhance my reputation as a competent and attentive healthcare provider. | 23 (21) | 53 (48) | 25 (23) | 9 (8) | 1 (1) |
| EE | EE1 | My interaction with the continuous monitoring and deterioration alerting system is clear and understandable. | 23 (21) | 74 (66) | 12 (11) | 2 (2) | 0 (0) |
|  | EE2 | It is easy for me to become skilled at using the continuous monitoring and deterioration alerting system. | 22 (20) | 65 (59) | 18 (16) | 5 (5) | 1 (1) |
|  | EE3 | I find the continuous monitoring and deterioration alerting system easy to use. | 22 (20) | 67 (60) | 18 (16) | 4 (4) | 0 (0) |
|  | EE4 | Learning to operate the continuous monitoring and deterioration alerting system is easy for me. | 30 (27) | 63 (57) | 14 (13) | 4 (4) | 0 (0) |
| SI | SI1 | People who influence my behaviour think that I should use continuous monitor with deterioration alerting system. | 9 (8) | 55 (50) | 35 (32) | 12 (11) | 0 (0) |
|  | SI2 | People who are important to me think that I should use the continuous monitoring and deterioration alerting system. | 12 (11) | 41 (37) | 46 (41) | 12 (11) | 0 (0) |
|  | SI3 | The hospital administration has helped promote the use of the continuous monitoring and deterioration alerting system. | 17 (15) | 53 (48) | 29 (26) | 11 (10) | 1 (1) |
|  | SI4 | In general, the organization has provided adequate support for the implementation and use of the continuous monitoring and deterioration alerting system. | 16 (14) | 58 (52) | 29 (26) | 7 (6) | 1 (1) |
| FC | FC1 | I have the necessary resources to effectively use the continuous monitoring and deterioration alerting system. | 19 (17) | 62 (56) | 16 (14) | 14 (13) | 0 (0) |
|  | FC2 | I have the necessary knowledge and skills to use the continuous monitoring and deterioration alerting system. | 24 (22) | 68 (61) | 16 (14) | 3 (3) | 0 (0) |
|  | FC3 | The continuous monitoring and deterioration alerting system is compatible with other systems I use in my work. | 11 (10) | 59 (53) | 24 (22) | 15 (14) | 2 (2) |
|  | FC4 | Adequate support is available to me in case I face any difficulties while using the continuous monitoring and deterioration alerting system (such as device malfunctions). | 9 (8) | 72 (65) | 27 (24) | 3 (3) | 0 (0) |
| BI | BI1 | I intend to use the continuous monitoring and deterioration alerting system in the coming months. | 25 (23) | 55 (50) | 23 (21) | 4 (4) | 4 (4) |
|  | BI2 | I predict that I will use the continuous monitoring and deterioration alerting system in the coming months. | 29 (26) | 56 (51) | 16 (14) | 7 (6) | 3 (3) |
|  | BI3 | I plan to make use of the continuous monitoring and deterioration alerting system in the coming months. | 28 (25) | 48 (43) | 27 (24) | 6 (5) | 2 (2) |

**Table S4 Measurement model diagnostics (reflective composites)**

*Cronbach’s α ≥0.70, CR ≥0.70, AVE ≥0.50 indicate acceptable reliability/convergent validity; √AVE should exceed inter-construct correlations (Fornell–Larcker). HTMT <0.85–0.90 indicates discriminant validity.* *Cronbach’s α can be biased for k=2. We therefore report CR (0.768) and AVE (0.623); inter-item correlation available on request.*

| Construct | Cronbach α | Composite Reliability (CR) | AVE | √AVE | Min loading | Max loading |
| --- | --- | --- | --- | --- | --- | --- |
| PE | 0.779 | 0.859 | 0.604 | 0.777 | 0.679 | 0.839 |
| EE | 0.725 | 0.83 | 0.55 | 0.742 | 0.704 | 0.814 |
| SI | 0.599 | 0.771 | 0.46 | 0.678 | 0.555 | 0.765 |
| FC | 0.66 | 0.798 | 0.499 | 0.706 | 0.603 | 0.781 |
| BI | 0.939 | 0.961 | 0.891 | 0.944 | 0.942 | 0.947 |
| Use Behaviour | 0.395 | 0.768 | 0.623 | 0.789 | 0.789 | 0.789 |

**Table S5 HTMT matrix (constructs)**

|  | PE | EE | SI | FC | BI | UB |
| --- | --- | --- | --- | --- | --- | --- |
| PE | 1.0 | 0.576 | 0.583 | 0.402 | 0.357 | 0.288 |
| EE | 0.576 | 1.0 | 0.536 | 0.714 | 0.28 | 0.163 |
| SI | 0.583 | 0.536 | 1.0 | 0.721 | 0.455 | 0.472 |
| FC | 0.402 | 0.714 | 0.721 | 1.0 | 0.599 | 0.144 |
| BI | 0.357 | 0.28 | 0.455 | 0.599 | 1.0 | 0.427 |
| Use Behaviour | 0.288 | 0.163 | 0.472 | 0.144 | 0.427 | 1.0 |

**Table S6 Inner model collinearity (VIF)**

| Predictor (Behaviour Intention) | VIF |
| --- | --- |
| PE | 1.35 |
| EE | 1.37 |
| SI | 1.31 |
| Voluntariness of Use | 1.19 |
| Age | 1.23 |
| Gender | 1.09 |
| Use Experience | 1.12 |
| Predictor (Use Behaviour) | VIF |
| BI | 1.29 |
| FC | 1.31 |
| Age | 1.09 |
| Use Experience | 1.08 |

Inner-model VIFs support a conservative common-method screen (<3.3).

**Table S7 Interview participant characteristics and CM-DAS usage patterns (n = 10)**

| Number | Professionals | Department | Gender | Age (years) | Type of device | Use experience | Voluntariness of Use | Use Behaviour in past 12 months |
| --- | --- | --- | --- | --- | --- | --- | --- | --- |
| P1 | Nurse | Obstetrics and Gynaecology | Female | 30-39 | Wearable Device & Bedside Monitor | 2-5 year | Very Willing | Frequently (multiple times a week) |
| P2 | Nurse | Obstetrics and Gynaecology | Female | 30-39 | Wearable Device & Bedside Monitor | 10 years above | Very Willing | Daily |
| P3 | Nurse | Obstetrics and Gynaecology | Female | 20-29 | Bedside Monitor | 2-5 year | Very Willing | Daily |
| P4 | Nurse | Mental Health | Female | 30-39 | Wearable Device & Bedside Monitor | 10 years above | Very Willing | Occasionally (once a week) |
| P5 | Nurse | Neurology | Female | 30-39 | Wearable Device & Bedside Monitor | 10 years above | Very Willing | Daily |
| P6 | Nurse | General Internal Medicine | Female | 20-29 | Bedside Monitor | Less than 1 year | Very Willing | Never |
| P7 | Physician | General Surgery | Female | 30-39 | Wearable Device | 2-5 years | Very Willing | Rarely (once a month or less) |
| P8 | Physician | Mental Health | Male | 20-29 | Bedside Monitor | 1-2 year | Neutral | Never |
| P9 | Nurse | Neurology | Female | 20-29 | Wearable Device & Bedside Monitor | 2-5 years | Willing | Frequently (multiple times a week) |
| P10 | Nurse Assistant | General Surgery | Female | 20-29 | Bedside Monitor | 1-2 years | Willing | Rarely (once a month or less) |
